# A case of delayed bleeding of the chest wall after VATS treated with transcatheter arterial embolization

**DOI:** 10.1093/jscr/rjae271

**Published:** 2024-05-12

**Authors:** Yohei Kameda, Hiroyuki Osawa, Yui Sueishi, Yoshihiro Ishikawa, Takamitsu Maehara

**Affiliations:** Department of Thoracic Surgery, Yokohama Minami Kyosai Hospital, 1-21-1, Mutsuurahigashi, Kanazawa-ku, Yokohama 236-0037, Kanagawa, Japan; Department of Thoracic Surgery, Yokohama Minami Kyosai Hospital, 1-21-1, Mutsuurahigashi, Kanazawa-ku, Yokohama 236-0037, Kanagawa, Japan; Department of General Thoracic Surgery, Yokohama City University Hospital, 3-9, Fukuura, Kanazawa-ku, Yokohama 236-0004, Kanagawa, Japan; Department of General Thoracic Surgery, Yokohama City University Hospital, 3-9, Fukuura, Kanazawa-ku, Yokohama 236-0004, Kanagawa, Japan; Department of Thoracic Surgery, Yokohama Minami Kyosai Hospital, 1-21-1, Mutsuurahigashi, Kanazawa-ku, Yokohama 236-0037, Kanagawa, Japan

**Keywords:** chest wall, delayed bleeding, lateral thoracic artery, VATS

## Abstract

We report a case of delayed bleeding after video-assisted thoracic surgery (VATS) that was successfully treated with transcatheter arterial embolization. An 81-year-old woman underwent a pleural biopsy via VATS for pleural dissemination of lung cancer. The postoperative course was good, but 8 days later she was hospitalized for swelling in the right axilla and was admitted to our hospital with a diagnosis of delayed postoperative hemorrhage. Gauze compression was performed, and the patient was discharged without exacerbation of hematoma. However, 4 days later, she was hospitalized for rapidly worsening swelling and pain. Chest computed tomography at the time of rebleeding showed an increase in the hematoma and extravasation in the peripheral right lateral thoracic artery. The patient was immediately treated with emergency angiography, and coil embolization was performed. After this treatment, the patient has done well and there has been no subsequent recurrence of bleeding.

## Introduction

Delayed chest wall bleeding is a rare complication of video-assisted thoracic surgery (VATS). We report a case of delayed bleeding after VATS that was successfully treated with transcatheter arterial embolization (TAE).

## Case report

**Figure 1:**
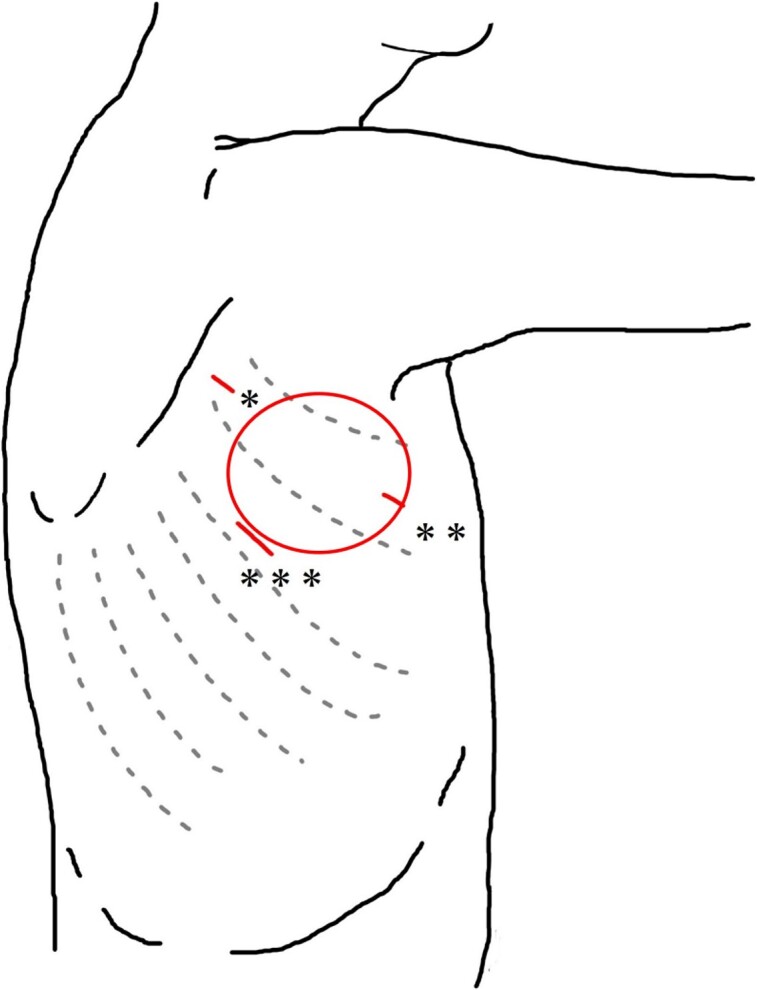
Schema of the surgical wound in VATS. ^*^Third intercostal space on the anterior edge of the scapula. ^*^^*^Third intercostal space on the midaxillary line. ^*^^*^^*^Fifth intercostal space on the posterior axillary line. Circle: Location of swelling.

An 81-year-old woman with suspected lung cancer of the right middle lobe with pleural dissemination underwent a pleural biopsy via VATS (Fig. 1). She also had chronic carotid artery stenosis and took clopidogrel, which was stopped 7 days before surgery. Before wound closure, hemostasis of the incision and port insertion site was confirmed by thoracoscopy. On the second day after surgery, she was discharged without any complications and oral administration of clopidogrel was resumed. Eight days later, she was hospitalized for swelling in the right axilla and was admitted to our hospital with a diagnosis of delayed postoperative hemorrhage (Fig. 1). Since her vital signs were stable and pain was mild, gauze compression was performed at night. Oral clopidogrel was discontinued. The next morning, the hematoma had not grown, so gauze pressure was continued for 2 days. She was discharged with no exacerbation.

**Figure 2:**
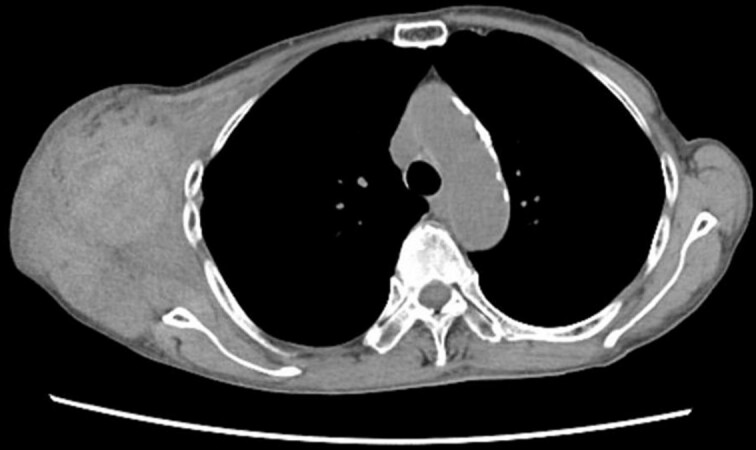
Chest CT at the time of the first bleeding showed formation of a hematoma on the right chest and front of the scapula.

**Figure 3:**
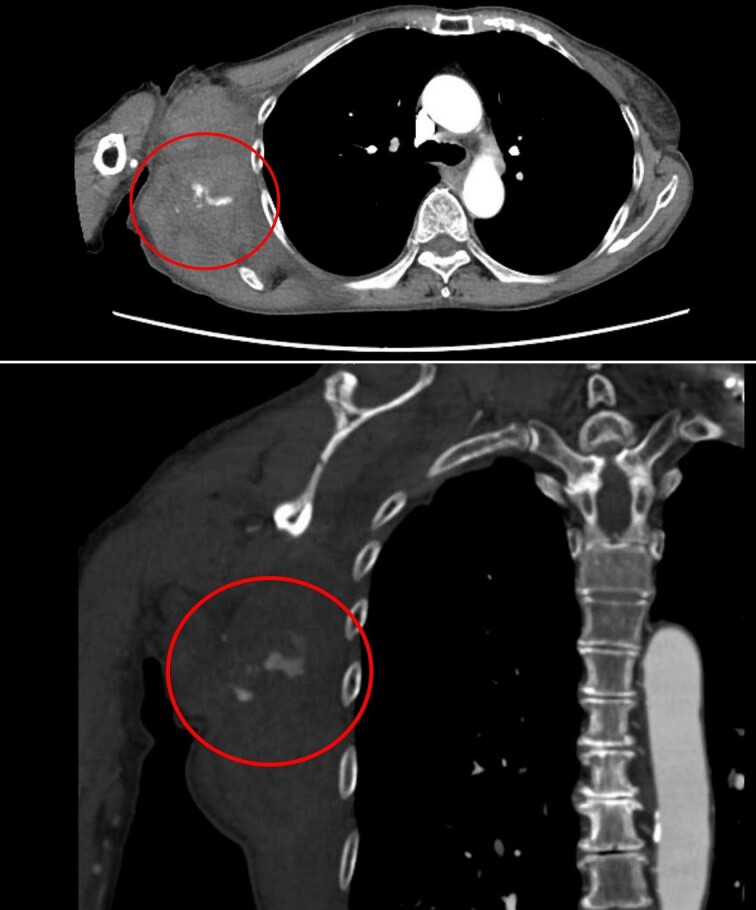
Contrast-enhanced CT at the time of the second bleeding showed growth of the hematoma and extravasation of contrast agent in a branch of the right lateral thoracic artery.

**Figure 4:**
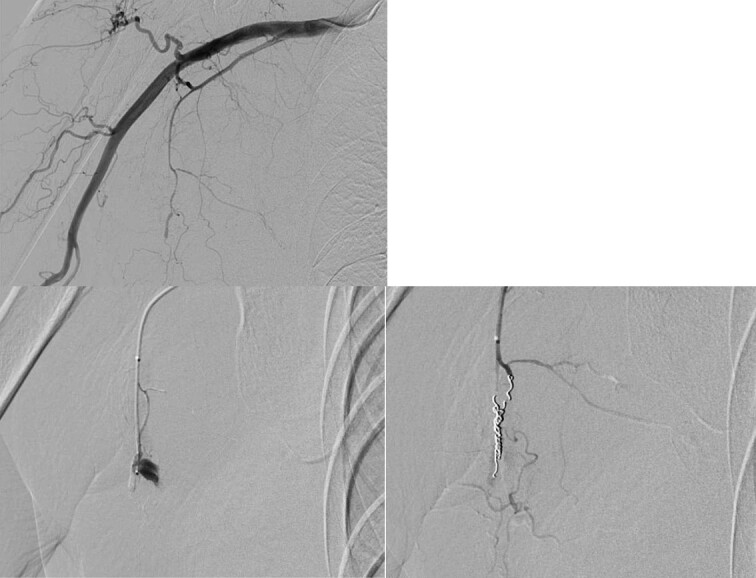
Angiography showed active bleeding from a pseudoaneurysm of the right lateral thoracic artery. Coil embolization was performed.

Four days later, the patient was hospitalized for rapidly worsening swelling and pain, and was readmitted with a diagnosis of rebleeding from the chest wall. Physical examination revealed a fist-sized subcutaneous mass on the right side of the chest, with severe pain. Laboratory data indicated anemia (Hb 7.8 g/dl) and no coagulation disorder: % prothrombin time (PT) 115.1%, prothrombin time-international normalized ratio (PT-INR) 0.93, and activated partial thromboplastin time 23.6 s. Computed tomography (CT) at the time of the first hemorrhage showed the hematoma on the right chest and anterior to the scapula (Fig. 2). Contrast-enhanced CT at the time of rebleeding showed an increase in hematoma and extravasation in the peripheral right lateral thoracic artery (Fig. 3). The patient was immediately treated with emergency angiography, which revealed active bleeding from a pseudoaneurysm of the right lateral thoracic artery. Thus, coil embolization was performed (Fig. 4). There were no TAE-related complications and the patient was discharged to home on the fifth hospital day. Oral clopidogrel was resumed and there has been no recurrence of bleeding.

## Discussion

Chest wall bleeding may occur due to blunt trauma [1] and after thoracentesis or insertion of a chest drainage tube [2–5]. Some cases only develop hematomas on the chest wall, while others develop hemothorax [6]. However, delayed bleeding of the chest wall after thoracic surgery is rare. There has been a report of a complication after valvular heart surgery with small thoracotomy [6], but complications after lung cancer surgery or thoracoscopic surgery have not been described. The lateral thoracic artery is a branch from the axillary artery that descends the chest wall and enters the serratus anterior muscle. Conventional thoracotomy such as posterolateral incision or axillary thoracotomy often involves dissecting this vessel, but the wide operative field allows sufficient hemostasis through ligation or cauterization. This may be one of the reasons why there are few case reports of delayed bleeding after thoracic surgery.

Use of VATS in general thoracic surgery is increasing, with the 2017 annual report of the Japanese Society of Thoracic Surgery finding use of this method in 73% of primary lung cancer surgeries and 94% of benign lung tumor surgeries [7]. VATS has been suggested to be superior or similar to conventional open surgery in terms of perioperative complication rates [8–11]. However, there are no large-scale studies comparing the incidence of delayed hemorrhage of the chest wall after VATS and thoracotomy. VATS has a smaller surgical field for treatment of the chest wall vasculature and the risk of bleeding may be higher.

In this case, when the operation was performed, the branch vessels of the lateral thoracic artery were coagulated and dissected immediately below the surgical wound, and hemostasis was obtained during the operation. However, 10 days after surgery, the patient developed delayed bleeding. This was thought to have been caused by the increase in body movement upon return to daily life after discharge and the resumption of oral antiplatelet drugs. Regarding the timing of delayed chest wall bleeding, Kamada *et al.* [6] found that hemothorax occurred 7 days after thoracic surgery, and Misthos *et al*. [12] reported a time from injury to onset of 2–14 days (mean 7 days) in 52 patients with delayed post-traumatic hemothorax. In this case, bleeding occurred 10 and 17 days after the operation. These findings suggest that careful follow-up for about 2 weeks is important for late complications after thoracoscopic surgery, especially in high-risk cases such as those of patients on anticoagulants and antiplatelet drugs.

As a method for hemostasis, we chose gauze compression for the initial postoperative bleeding, but this was insufficient and rebleeding occurred 17 days after the operation. Other treatments include hemostasis by endovascular intervention [6, 13–15] and surgical hemostasis. In this case, we chose endovascular treatment, since this is less invasive than reoperation, the bleeding site was identifiable on angiography, and risks of general anesthesia, wound infection and lung injury in reopening surgery were avoided. The outcome suggests that angiography may be a better treatment method, especially in cases with unstable vital signs.

We experienced a case of delayed hemorrhage of the chest wall after VATS. This case indicates that patients taking anticoagulants or antiplatelets should be carefully monitored after surgery, and that TAE is an effective method for hemostasis.

## References

[1] Dogrul BN , Kiliccalan I, Asci ES, Peker SC. Blunt trauma related chest wall and pulmonary injuries: an overview. Chin J Traumatol 2020;23:125–38. 10.1016/j.cjtee.2020.04.003. 32417043 PMC7296362

[2] Cantey EP , Walter JM, Corbridge T, Barsuk JF. Complications of thoracentesis: incidence, risk factors, and strategies for prevention. Curr Opin Pulm Med 2016;22:378–85. 10.1097/MCP.0000000000000285. 27093476 PMC8040091

[3] Matsuura K , Tada M, Sumi T, et al. Unexpected haemorrhage from lateral thoracic artery following the removal of a pleural drainage tube. Respirol Case Rep 2021;9:e0882. 10.1002/rcr2.882. 34849236 PMC8611179

[4] Schnell J , Beer M, Eggeling S, Gesierich W, Gottlieb J, et al. Management of spontaneous pneumothorax and post-interventional pneumothorax: German S3 guideline. Respiration 2019;97:370–402. 10.1159/000490179. 30041191

[5] Seki M , Yoda S. Unexpected massive hemorrhage following the removal of a pleural drainage tube. Intern Med 2015;54:953–4. 10.2169/internalmedicine.54.3719. 25876579

[6] Kamada K , Kitahara H, Koichi Y, et al. Delayed thoracic wall bleeding after minimally invasive mitral valve repair. J Surg Case Rep 2019;2019:1–3. 10.1093/jscr/rjz187. PMC6565816 31214324

[7] Shimizu H , Okada M, Tangoku A, et al. Thoracic and cardiovascular surgeries in Japan during 2017 annual report by the Japanese association for thoracic surgery. Gen Thorac Cardiovasc Surg 2020;68:414–49. 10.1007/s11748-020-01298-2. 32140991

[8] Wu N , Wu L, Qiu C, et al. A comparison of video-assisted thoracoscopic surgery with open thoracotomy for the management of chest trauma: a systematic review and meta-analysis. World J Surg 2015;39:940–52. 10.1007/s00268-014-2900-9. 25446488

[9] Bendixen M , Jørgensen OD, Kronborg C, et al. Postoperative pain and quality of life after lobectomy via video-assisted thoracoscopic surgery or anterolateral thoracotomy for early stage lung cancer: a randomised controlled trial. Lancet Oncol 2016;17:836–44. 10.1016/S1470-2045(16)00173-X. 27160473

[10] Inderbitzi RG , Grillet MP. Risk and hazards of video-thoracoscopic surgery: a collective review. Eur J Cardiothorac Surg 1996;10:483–9. 10.1016/S1010-7940(96)80412-X. 8855418

[11] Solaini L , Prusciano F, Bagioni P, et al. Video-assisted thoracic surgery (VATS) of the lung: analysis of intraoperative and postoperative complications over 15 years and review of the literature. Surg Endosc 2008;22:298–310. 10.1007/s00464-007-9586-0. 17943372

[12] Misthos P , Kakaris S, Sepsas E, et al. A prospective analysis of occult pneumothorax, delayed pneumothorax and delayed hemothorax after minor blunt thoracic trauma. Eur J Cardiothorac Surg 2004;25:859–64. 10.1016/j.ejcts.2004.01.044. 15082295

[13] Fujihara T , Itoh N, Yamamoto S, Kurai H. Lateral thoracic artery aneurysm with lung abscess and empyema caused by Streptococcus intermedius. J Gen Fam Med 2021;22:296–7. 10.1002/jgf2.448. 34485000 PMC8411411

[14] Pontell M , Scantling D, Babcock J, et al. Lateral thoracic artery pseudoaneurysm as a result of penetrating chest trauma. J Radiol Case Rep 2017;11:14–9. 10.3941/jrcr.v11i1.3015. PMC5443627 28580065

[15] Lee TH , Park YS, Chung DJ, et al. Spontaneous rupture of the lateral thoracic artery in patients with liver cirrhosis. Korean J Intern Med 2008;23:152–5. 10.3904/kjim.2008.23.3.152. 18787369 PMC2686960

